# Impact of extended course duration and stricter study organization on attrition and academic performance of medical students

**DOI:** 10.3325/cmj.2013.54.192

**Published:** 2013-04

**Authors:** Roberta Andrea Tešija, Silvija Maslov Kružičević, Adriana Banožić, Carlos David Esteban, Damir Sapunar, Livia Puljak

**Affiliations:** Department of Anatomy, Histology and Embryology, University of Split School of Medicine, Split, Croatia

## Abstract

**Aim:**

To assess whether extended medical school duration, block/modular structure of subjects, not allowing students to transfer exams into the higher course year, and curriculum implementation in line with the Bologna Accord are associated with lower attrition and better academic outcomes of medical students.

**Methods:**

We retrospectively investigated curricula at the University of Split School of Medicine and academic outcomes of 2301 medical students during a 33-year period (1979-2011). The following data were obtained: grade point average (GPA) at the end of the studies, duration of studies, graduation on time, and whether the student graduated or not.

**Results:**

After extension of medical curriculum from 5 to 6 years, students had significantly better grades (3.35 vs 3.68; *P* < 0.001), shorter study duration (7.0 vs 6.0 years; *P* < 0.001), and more students graduated on time (6.5% vs 57%; *P* < 0.001). Changes in the 6-year curriculum, such as stricter study regulations and adoption of Bologna Accord, were associated with better indicators of students’ academic success. The lowest attrition and the highest grades during the studied period were observed after the implementation of the Bologna Accord in 2005.

**Conclusion:**

Introduction of a longer medical curriculum, block/modular subject structure, stricter regulations of exam transfer, and curriculum in line with the Bologna Accord may contribute to better academic outcomes and lower attrition of medical students.

The prototype medical curriculum in Europe is a first-degree course that lasts 5-7 years, with basic science subjects in the first years and clinical subjects in the later years, whereas in the USA it is a 4-year postgraduate course (1). A study published in 2003 showed that curricula in European medical schools greatly differed in content and duration (2). Rigorous evaluation of curriculum reform and educational interventions are necessary if we want to ensure that we are providing the best possible education to our students (1).

When the New Pathway curriculum was introduced at Harvard Medical School, no difference was found in problem-solving skills or biomedical knowledge base between students in the traditional and new curriculum. However, students in the new curriculum learned differently, acquired specific knowledge, skills, and attitudes, and had a more satisfying and challenging medical school experience without losing biomedical competence (3).

Curriculum changes are often based on content modification, without changing the fundamental principles (4). While personal factors influencing attrition and academic success have been extensively studied, analyses of the impact of curricular changes on these variables are sparse. Iputo and Kwizera (5) have shown that after the introduction of an innovative problem-based learning curriculum in South Africa, student attrition was reduced from 23% to 10%, the percentage of students who graduated on time within 6 years increased from 55% to 67%, and average study duration in the innovative cohort was reduced from 6.7 to 6.4 years (5).

The University of Split School of Medicine (USSM) in Split, Croatia was established in 1997, after separating from the Medical School in Zagreb due to difficulties stemming from the organizational scheme of work, as well as complex political and geographical issues (6). Founding of the independent School enabled the development of faculty and research capacities, which could have influenced the quality of teaching (7-9). Since then, the USSM underwent several curriculum changes. The type of curriculum is mostly defined either according to the organization of the content or the instructional method (10). At the USSM, instructional method remained the same during the 33 analyzed years, while curriculum duration, content, and study regulations underwent several changes. The last change was the implementation of the Bologna Accord. The 1999 Bologna Accord initiated a transformation process of higher education in Europe by promoting a flexible approach, curriculum review, and implementation of a more student-focused approach and new quality procedures (11).

The aim of this article was to study the impact of curriculum extension and study rules change on attrition and academic outcomes of medical students in a medical school that underwent several curriculum changes. The hypothesis was that extension of the course duration, block/modular structure of subjects, not allowing students to enroll in the higher course year without passing all the exams from the previous year, and curriculum in line with the Bologna Accord were associated with lower attrition and better academic outcomes of medical students.

## Methods

### Study design

We retrospectively studied academic records of all students (N = 2301) who studied medicine in Split, Croatia during a 33-year period from 1979 to 2011. Data were collected at the student registry and the School archives, and the study was approved by the Ethics Committee of the USSM.

### Setting

Medical studies in Split started enrolling first-year students in 1979. Students in Croatia apply for university studies, including medical school after having completed 8-year elementary school and 4-year high school.

Curriculum duration was changed in the academic year 1989/1990, when a 5-year curriculum was extended to 6 years. In 1997, the school's university affiliation was changed. Initially, the School was a branch of University of Zagreb School of Medicine and students were allowed to enroll in a new/higher year without passing all the exams. When the USSM was founded and much stricter regulations were introduced, students were required to pass all the exams before enrolling in a higher course year and the content was taught in a block/modular manner (6).

In 2005, the curriculum at the USSM was changed in line with the regulations of the Bologna Accord – the block/modular delivery of subjects was kept and students were again allowed to enroll in a higher course year without having to pass all the exams, but the number of times that students were allowed to take one exam before losing the right to study was limited to 8. Before 2005, there were no such restrictions – students were allowed to take an exam for an unlimited number of times. The grade system remained the same throughout the USSM history.

### Data collection

The following data were obtained: grade point average (GPA) at the end of the studies, duration of studies, and data on students’ graduation on time. To get the number of students who graduated on-time, we obtained the number of students who graduated within 5 years during the 5-year curriculum and who graduated within 6 years in the 6-year curriculum. Additionally, the number of students who graduated within 6 years in the 5-year curriculum was obtained. Annual attrition was calculated as the median number of students who were lost per year during different analyzed periods.

Students were classified into groups according to the curriculum that they were enrolled into and the enrolment year. According to the curriculum, students were classified into the 5-year (1979-1989) and the 6-year curriculum (1990-2011). The 6-year curriculum was further divided, based on organizational changes, into the subsidiary Zagreb period (1990-1996), in which the School was a branch of University of Zagreb School of Medicine; independent pre-Bologna (1997-2004), from the year the School became independent to the introduction of Bologna curriculum; and the period after the implementation of the Bologna Accord (2005-2011).

### Statistical analysis

Data analysis was performed with SPSS 15.0 (SPSS Inc., Chicago, IL, USA). Descriptive statistics data are expressed as median and interquartile range (IQR). Differences between groups were analyzed using Mann-Whitney test, χ^2^ test, and Kruskal-Wallis test. Normality of data was tested using Kolmogorov-Smirnov test. Statistical significance was set at *P* < 0.05.

## Results

### Overall academic success and attrition at the School

We studied 33 successive cohorts of medical students who were enrolled in the School from 1979 to 2011. Of 2301 enrolled students, 1284 (56%) graduated, 641 in the 5-year curriculum and 643 in the 6-year curriculum. There were 446 men (35%) and 838 women (65%) (*P* = 0.017). Median (IQR) graduation GPA was 3.68 (0.68) and the study duration of graduated students was 7.0 (2.0) years. Median (IQR) graduation GPA of men was significantly lower than that of women (3.64 [1.77] vs 3.71 [1.30], *P* = 0.020). The length of studying of both women and men was 7.0 (2.0) years, showing no significant differences (*P* = 0.700). The minimum and maximum study duration was 5 and 25 years, respectively.

Of the remaining 1017 students, 438 (43%) were still studying at the beginning of 2012, and their curriculum was in line with the Bologna Accord. Total attrition rate was 25% of enrolled students (579/2301). Among dropout students there were significantly more women (235 men or 41% vs 344 women or 59%, *P* < 0.001). Dropout students spent a median (IQR) of 3.0 (5.0) years at the School before leaving.

### Curriculum duration and academic outcomes

Median study duration of graduates in the 5-year curriculum was significantly longer than of graduates in the 6-year curriculum (7.0 vs 6.0 years, *P* < 0.010) (Table 1). There was no significant difference in average GPA between students in the 5-year and 6-year curriculum (Table 1). Significantly more students graduated on time after the curriculum was extended from 5 to 6 years (Table 1). In the 5-year curriculum, 28% of students (179/623) graduated within 6 years, which is significantly lower than 57% who graduated in the 6-year curriculum (χ^2^ = 122.5, *P* < 0.001). The annual attrition was significantly lower in the 6-year than in 5-year curriculum. On average, the 6-year curriculum had 882 more teaching hours than 5-year curriculum (Table 1).

**Table 1:** Academic outcomes in the 5-y and 6-y curriculum of medical studies in Split

	Curriculum		
	5-y (1979-1989)	6-y (1990-2011)	*P*
Teaching hours, N	4500	5382*	
Grade point average, median (IQR)	3.35 (1.1)	3.68 (1.63)	<0.010
Study duration of individual students, median (IQR)	7.0 (2.0)	6.0 (1.0)	<0.001
Graduating on-time, n/N (%)	42/641 (6.5)	365/643 (57.0)^†^	<0.001
Annual attrition, median (IQR)	18 (6.8)	10 (6.0)	0.080

*Average of 3 different curricula from 1990-2011.

†For 8 students the data on study duration were not available.

### Study regulations and academic outcomes

The length of studying (7.79 vs 6.54 years, *P* < 0.001) and GPA (3.67 vs 3.83, *P* < 0.001) significantly improved after the School became independent (1997-2008) compared to the period when the School was not an independent institution (1979-1996).

However, since the “dependent” period included 10 years of the 5-year curriculum, we made detailed analyses of different programs of the 6-year curriculum. All indicators of academic success improved in each subsequent period (Table 2). Compared to the “dependent” period, average students’ GPA increased and average study duration decreased in the period when the School introduced stricter course regulation. Additionally, average annual number of dropouts was reduced from 25 to 15 (Table 2).

**Table 2:** Academic outcomes in three different periods of a 6-y curriculum of medical studies in Split

	Curriculum			
	Initial period (1990-1996)	Intermediate period (1997-2004)	Bologna curriculum (2005-2011)	*P*^†^
Teaching hours, N	5400	5580	5167	
Grade point average, median (IQR)	3.50 (0.67)	3.80 (0.57)	4.00 (0.93)	<0.001
Study duration, median (IQR)	7 (2.0)	6 (1.0)	6 (0.0)	<0.001
Graduating on-time, n/N (%)	126/265 (48.0)	195/314 (62.0)	44/64 (69.0)*	0.150
Annual attrition, median (IQR)	21 (5.5)	18.5 (10.0)	15 (3.5)	0.600

*Percentage of students who graduated on time in the Bologna curriculum was calculated out of number of students who enrolled in the University of Split School of Medicine in 2005 and did not leave the School by the end of the 2011.

†Kruskal-Wallis test.

## Discussion

This study found better academic outcomes and lower attrition of medical students after the curriculum was extended and in the periods marked with more stringent study regulations. The best academic outcomes, analyzed through student attrition, average grades on graduation, and study duration, were observed in the most recent period, in which the curriculum was organized in line with the Bologna Accord.

The study duration was significantly reduced after the curriculum was extended from 5 to 6 years. One-year curriculum extension was also associated with a significantly larger number of students who graduated on time (6% vs 59%). The number of students who graduated within 6 years was significantly lower during the 5-year curriculum than during the 6-year curriculum, which could indicate that the introduction of an additional course year was beneficial for students’ academic performance.

In an effort to achieve efficient education, there are proposals for reducing training time and cutting costs. However, professional development requires that the individual is given time to mature. Reducing training time and the number of clinical rotations may be detrimental before we find out how we should design curricula according to the new competence and outcome paradigms (12).

Stringent regulations on the higher year enrollment and the number of times students are allowed to take an exam were associated with better students’ academic outcomes. Our previous study in the same institution showed that students who were enrolled later in the School’s history had a higher likelihood of graduating (13). This study made a step further and analyzed various curricula at the USSM.

Improvement in academic outcomes in our case may be partly explained with the introduction of a block/modular structure of curriculum, since it has been shown that this kind of curriculum is linked with an increase in the average grades (14). A positive impact of curricular changes in the independent USSM was reported already in 2001, when new regulations were associated with lower attrition and better academic outcomes, compared to the previous period, in which subjects were taught in a longitudinal structure and students were allowed to enroll in a higher course year even if they had not pass all the exams from the previous year (15).

Better grades observed later in the history of the USSM could be a result of grade inflation. Bergovec et al (16) found an increasing trend of the GPA of 2861 students from 9 representative generations at the University of Zagreb School of Medicine from 1920 to 1990.

An increase in grades and grade inflation has been described abroad as well (17,18). Since employment and professional advancement may depend on the grades acquired during the studies, students have been reported to ask teachers to give them better grades than they deserved and teachers have been reported to comply (17).

In this study, there could have been other interfering variables, such as students’ sex. A slow but steady increase in the number of women and their better performance could have potentially contributed to the findings. Our recent study at the same school showed that the proportion of enrolled female students increased from 52% in 1979 to 77% in 2005, and that female graduates had a significantly higher average grade when graduating (7). However, although significant, this difference in graduation grades was not dramatic, because the mean graduation GPA was 3.62 for men and 3.69 for women (7). Furthermore, we did not observe any significant difference in study duration between women and men, and women made 59% of our dropout students. Therefore, we could conclude that sex differences are not a significant interfering variable.

The curriculum content was changed several times throughout the School’s history, as well as the total number of teaching hours. Extensive changes in medical education have been recently observed in other parts of the world too, and they need to be assessed to estimate the reforms’ success (19). There is little evidence, however, on the impact of various curricula and educational policies on students’ learning if one takes into the account previous students’ grades and differences between students. The few studies that have systematically evaluated the impact of various curricula have failed to show any substantial differences between them (20). Therefore, we believe that what made a difference were the structural changes rather than the curriculum content itself.

At the moment, there are not enough data to fully comprehend the effect of the Bologna study regulations, as the students studying under this regimen are just starting to graduate, but we can already observe that the Bologna curriculum has significantly reduced student attrition, as it was reported previously (5).

Attrition is an important indicator of university performance. Students who do not graduate represent a waste of resources and loss for the labor market, as they cannot use what they have gained during their studying (21). The attrition rate of 25% represents a waste of time and resources. Compared to the previous report from the same school, the overall attrition decreased, owing to the good results in the later periods of the school’s history (13). Other studies showed lower attrition rates. Attrition at the Leeds School of Medicine over the 10 years was 14% (22). In an American study, it was less than 4% and about 80% of students graduated on time (23). A possible reason for higher rates in Croatia may be that most of Croatian students do not pay tuition (13).

Curricular changes in our School have been beneficial for students’ outcomes, but there is still room for improvement. Once students are admitted, there should be instruments to identify students at risk for academic failure. Our previous study showed that 50% of the attrition happened during the freshmen year, so an interview with a student counselor could be scheduled for all students after the first semester to identify and support those in risk for attrition (13). Other effective interventions for students at risk have been previously described (24,25).

Shorter study duration and better grades do not necessarily equal better education. Our results could be complemented with the assessment of some other criteria, such as clinical skills. Furthermore, our analyses have been conducted over a long period of time, during which there was a war in Croatia. Therefore, it is likely that there are some other confounding variables that could have influenced the results.

In conclusion, introduction of a longer medical curriculum, block/modular subject structure, and stricter regulations regarding the transfer of exams were associated with better academic outcomes and lower attrition. Bologna-based curriculum was linked with lower attrition compared to previous curricula.
